# The relationship between cognitive emotion regulation strategies and emotional manipulation among health colleges students: a cross-sectional correlational study

**DOI:** 10.3389/fpsyt.2024.1469527

**Published:** 2024-09-23

**Authors:** Mai B. Alwesmi, Rasha Mohammed Bayounes, Norah Nasser Binrushaydan, Maha Ahmad Alanazi, Raghad Mohamed Salem, Raghad Ahmad Alomairi, Amal Zaid Albugami, Ebtisam Mohammed Alzahrani, Lama A. Alahmari, Naglaa Youssef

**Affiliations:** ^1^ Department of Medical-Surgical Nursing, College of Nursing, Princess Nourah bint Abdulrahman University, Riyadh, Saudi Arabia; ^2^ College of Nursing, Princess Nourah bint Abdulrahman University, Riyadh, Saudi Arabia

**Keywords:** cognitive emotion regulation, emotional manipulation, emotional intelligence, students, health colleges

## Abstract

Emotional intelligence is commonly associated with career success. Employees with higher emotional intelligence tend to reap greater benefits. However, emotional manipulation has been reported as a dark side of emotional intelligence, which refers to the self-perceived ability to control the emotions and actions of others for self-benefit. Healthcare professionals with high emotional intelligence often improve the overall patient experience. However, their ability to manipulate emotions can have a detrimental impact on the quality of treatment. This cross-sectional correlational study assessed cognitive emotion regulation strategies (CER) and emotional manipulation among students of health colleges. Two instruments were used to collect the data: 1) the emotional manipulation scale and 2) the cognitive emotion regulation questionnaire. A total of 362 students from health colleges responded to the questionnaire. The students had a moderate tendency to use emotional manipulation (25.09 ± 6.79 out of 50). The most frequently used CER strategies included adaptive strategies such as positive reappraisal with a mean score of 7.45 ± 2.15 out of 10 and maladaptive strategies such as rumination with a mean score of 7.33 ± 2.23 out of 10. The emotional manipulation score had a small but statistically significant negative correlation with two adaptive CER strategies: positive refocusing (r = -0.146, p = 0.005) and focus on planning (r = -0.144, p = 0.006). This study sheds light on the relationship between CER strategies and emotional manipulation, suggesting that poor use of adaptive strategies is associated with emotional manipulation. This finding highlights the importance of designing interventional programs that improve the ability of health colleges students to regulate their cognitive and emotional responses, thus improving their overall well-being and performance as future healthcare workers.

## 1 Introduction

Reliance on foreign healthcare professionals poses challenges in terms of cultural and linguistic alignment, continuity of care, and national healthcare security. The need to localize healthcare workers in Saudi Arabia to 60% from the current figure is a pressing issue, as the present statistics reveal significant gaps in the local workforce (1). Currently, the percentage of Saudis in the healthcare sector is 42% of physicians, 53% of dentists, 43% of nurses, 41% of pharmacists, and 84% of allied health personnel, which is a total of nearly 44% of healthcare workers (2). In response, there has been a concerted effort to increase the number of health colleges and students in these programs.

Emotional intelligence and cognitive emotional regulation (CER) are crucial for health college students, as they enhance their ability to communicate effectively with patients, fostering trust and understanding. By developing these skills, students can make shared ethical decisions that prioritize patient safety and care quality, mitigating the impact of emotional manipulation in clinical settings (3). Emotional intelligence is the ability to understand and perceive one’s own and others’ emotions, as well as the ability to use that information to guide decision-making and social communication (4). It has become a key aspect of the healthcare business to gain a competitive advantage over competitors in a highly dynamic environment where stakeholder satisfaction is the focus (5). People with high emotional intelligence often strategically use their ability to advance their own interests in organizational environments, even at the expense of others (5). More specifically, it can have disastrous consequences when clever managers use their skills to achieve what they want at the expense of other people and the company, which is evident in the healthcare sector (6). Although emotional intelligence tends to improve the optimal patient experience, emotional manipulation tends to negatively impact patients’ quality of life.

Emotional manipulation is a dark side of emotional intelligence, which refers to the self-perceived ability to control the emotions and actions of others for self-benefit (7). Several studies have reported that emotional manipulation is positively correlated with the emergence of conflict among colleagues, interference with colleagues’ interests, and counterproductive work behavior (8, 9). Healthcare workers are victims of a wider range of emotional manipulation and manipulative behaviors. Patients have often been shown to emotionally force doctors and nurses to make decisions that hinder the safety and quality of care offered (10).

Emotional manipulation is best described in terms of two constructs: prosociality and non-prosociality. The prosociality construct exists when emotional manipulation is intentional, voluntary, and beneficial to others or society as a whole (11). Non-prosociality, on the other hand, is described as that which serves to fulfill selfish interests and personal gains at the expense of other people, the organization and society as a whole (12). Self-reported skill-based emotional intelligence is positively correlated with prosocial and non-prosocial emotional manipulation (13), which is considered an aspect of interpersonal emotion regulation that is used to fulfill personal motivations (7, 14).

Cognitive emotional regulation is the conscious cognitive way of regulating emotions or modifying situations to reduce or prevent the sense of being overwhelmed (15).

The CER questionnaire was developed to identify strategies that individuals can use when experiencing a negative situation or event (16). Nine CER strategies were found to account for a large variance not only in negative situations but also in emotional relationships. These strategies have been classified into adaptive and maladaptive strategies (17). The adaptive strategies include acceptance, positive reappraisal, positive refocusing, refocusing planning, and perspective assessment, while the maladaptive strategies include self-blame, catastrophizing, rumination, and other blame.

Adaptive and maladaptive CER strategies have been linked to the development and maintenance of psychopathology (18). Both the absence of adaptive strategies and the presence of maladaptive strategies have been reported to place an individual at increased risk for emotional manipulation and psychopathology (19). Furthermore, difficulties with emotion regulation have shown a more positive association with maladaptive behaviors (16). Adaptive functions, including the ability to facilitate decision making, often required regulation and showed a positive association with well-being, while showing a negative association with psychopathology (20). Moreover, a significant association was found between cognitive strategies and symptoms of anxiety and depression (21). This poses an effect on how certain individuals relate to emotional manipulation abilities in either impulsivity or distress tolerance.

Much has remained unexplored with a growing interest in understanding CER strategies in relation to emotional manipulation abilities. The importance of CER strategies becomes more evident for healthcare workers to respond emotionally to their patients. The students of health colleges are future healthcare professionals. Their tendency to emotionally manipulate others, as well as their ability to use CER strategies, can negatively impact the work environment, their performance, and the performance of other healthcare workers.

Therefore, this study aimed to identify the frequently used CER strategies and assess the tendency toward use emotional manipulation and the relationship between them among students from health colleges.

## 2 Methods

### 2.1 Design and setting

This cross-sectional correlational study was conducted among students at health colleges of a large public university in Riyadh City, Saudi Arabia, in the period from 28 February 2024 to 19 March 2024.

### 2.2 Study population and sample

The sample size was calculated twice prior to conducting this study. The first calculation used the total population of health colleges within the selected university, which was 5539. The sample size was calculated using Raosoft (Raosoft) with the assumption of a 95% confidence interval, a 5% margin of error, and a 50% response distribution, giving a sample size of 360 students. The sample size was also calculated considering the power of the sample, since it is an ideal method for controlling type I and type II errors (22). Using G*power software with a power of 0.95, a medium effect size of 0.3, and an alpha error of 0.05 at two-tailed indicated that the required sample should be 138 to find a significant correlation. A convenient nonprobability sampling technique was used to recruit the required sample.

### 2.3 Instruments

An electronic survey, comprising two standardized scales alongside demographic characteristics data (i.e., college, major, age, grade point average (GPA), emotional intelligence training, and mental health status), was used to collect the study data.

#### 2.3.1 The emotional manipulation scale

The 10-item emotional manipulation scale by Austin et al. (7) was used to assess emotional manipulation. Participants responded to the following statements on a 5-likert scale, using “strongly disagree = 1” and “strongly agree = 5” as endpoints: 1) “I know how to embarrass someone to stop them from behaving in a particular way.”; 2) “I know how to make another person feel uneasy.”; 3) “I know how to play two people off against each other.”; 4) “I know how to make someone feel ashamed about something that they have done to stop them from doing it again.”; 5) “I know how to ‘wind up’ my close family and friends.”, 6) “I can use my emotional skills to make others feel guilty.”; 7) “I can make someone feel anxious so that they will act in a particular way.”; 8) “I can pay someone compliments to get in their ‘good books.”; 9) “I am good at reassuring people so that they’re more likely to go along with what I say” and 10) “I sometimes pretend to be angrier than I really am about someone’s behavior in order to induce them to behave differently in future.”. The total score ranges from 10 to 50 with a higher score indicating a higher tendency to use emotional manipulation. The tool has shown excellent internal reliability with Cronbach’s α = 0.88 (7).

#### 2.3.2 The cognitive emotion regulation questionnaire

The CER Questionnaire is a self-report assessment tool that can measure adults’ and adolescents’ cognitive coping mechanisms (23). The original instrument consists of 36 items (4 items in each subscale) in a nine-factor model: 1) Self-blame; 2) Acceptance; 3) Rumination; 4) Positive Refocusing; 5) Refocus on Planning; 6) Positive Reappraisal; 7) Putting into Perspective; 8) Catastrophizing; and 9) Other-blame (23). The short version of the CER Questionnaire, consisting of 18 items (2 items in each subscale), was used in our study (24). Participants rate their usage of specific cognitive coping strategies using a five-point Likert scale: (1) “(almost) never,” (2) “sometimes,” (3) “regularly,” (4) “often,” or (5) “(almost) always.” The score is calculated for each of the nine factors. Each factor in the short version includes two items that give a total score of 2 (never used) to 10 (frequently used cognitive coping method). The alpha coefficients for each subscale ranged from 0.68 to 0.89 (24).

### 2.4 Data collection

After obtaining ethical approval, the electronic survey was administered to five students of health colleges to assess its clarity, application, and feasibility before collecting the study data. The students verified the clarity of the survey and no changes were necessary. The questionnaire was subsequently uploaded to Google Docs and distributed by email to students at all health colleges in the selected university. Subsequently, additional reminders were sent until the desired sample size was reached.

### 2.5 Ethical approval

The study received ethical approval from the IRB. Ethical considerations were followed throughout the investigation, including the voluntary invitation of the participants and the reminder that they can withdraw their consent at any time. The data were anonymized, stored securely, and coded on the computer belonging to the first author. Participants should give their consent to participate by clicking on the ‘‘Yes’’ option before proceeding and accessing the study survey.

### 2.6 Statistical analysis

IBM SPSS Statistics version 26 was used to run the statistical analyses. Descriptive statistics such as means ± standard deviations, frequency, and percentages were used to describe the participants’ demographic data, CER, and emotional manipulation. The Kolmogorov-Smirnov test and the QQ plot were used to verify the normality of the data. Therefore, the Person correlation coefficient was utilized to investigate the relationship between CER and emotional manipulation. The p-value less than 0.05 was the threshold value at two-tailed. The internal reliability of the used scale was examined using Cronbach’s alpha with a cut-off value of more than 0.7 (25) as an internally reliable scale.

## 3 Results

### 3.1 Demographic characteristics

**Table 1** shows the demographic characteristics of the respondents, with a total sample size of 362 individuals. All the study participants were females (100%). The participants were from five health colleges (82.87%) and the foundation year (17.13%). The first to fourth academic year represented the most participants (89.77%). The largest age category in the study was 20-21 (49.9%). 44.6% of the participants reported very good academic performance, as represented by their GPA (4.49-3.75 out of 5). Finally, a self-reported history of mental health conditions revealed that 64 participants (17.6%) had a history of mental health conditions.

**Table 1:** Demographic characteristics.

Variable	Category	n.	%
Sex	Females	362	100%
Age	18-19	114	31.49
	20-21	181	50.00
	22-23	54	14.92
	24	13	3.59
College	Rehabilitation Sciences	68	18.78
	Dentistry	20	5.52
	Medicine	62	17.13
	Nursing	82	22.65
	Pharmacy	68	18.78
	Foundation Year for Health Sciences	62	17.13
Year of study	First	103	28.45
	Second	75	20.72
	Third	67	18.51
	Fourth	80	22.10
	Fifth	17	4.70
	Sixth	15	4.14
	Seventh	5	1.38
Academic Performance (grade point average)	Acceptable (2.74 - 2.00)	5	1.38
	Good (3.74 - 2.75)	47	12.98
	Very Good (4.49 - 3.75)	162	44.75
	Excellent (5.00 - 4.50)	148	40.88

### 3.2 Emotional manipulation

The mean score for emotional manipulation was 25.09 ± 6.80 (the total score ranges from 10 to 50), indicating a moderate level of emotional manipulation among the respondents (**Table 2**).

**Table 2:** Cognitive emotion regulation strategies and emotional manipulation.

Variable		Mean± SD	Pearson r^$^
Emotional Manipulation		25.09 ± 6.79	-
CER Strategies	Self-Blame^1^	6.51 ± 2.18	-0.007
	Acceptance^2^	7.11 ± 2.13	0.006
	Rumination^1^	7.33 ± 2.23	0.034
	Positive Refocusing^2^	5.97 ± 2.05	-0.146^**^
	Focus on Planning^2^	7.01 ± 2.18	-0.144^**^
	Positive Reappraisal^2^	7.45 ± 2.15	-0.054
	Putting into Perspective^2^	6.77 ± 2.26	0.003
	Catastrophizing^1^	6.21 ± 2.25	0.006
	Other-Blame^1^	4.70 ± 2.27	-0.010

^1^Maladaptive strategies.

^2^Adaptive strategies.

^$^Correlations between CER strategies and emotional manipulation.

^**^p < 0.005.

### 3.3 Cognitive emotional regulation strategies

CER strategies frequently used by the participants during negative events, as illustrated in **Table 2**. These are represented with a score range from 2 (never used) to 10 (frequently used). The adaptive strategy most frequently used was positive reappraisal with a mean score of 7.45 ± 2.15, while the least used adaptive strategy was positive refocusing with a mean score of 5.97 ± 2.05. On the other hand, the most frequently used maladaptive strategy was rumination with a mean score of 7.33 ± 2.23, while the least used maladaptive strategy was blame for the other with a mean score of 4.70 ± 2.27.

### 3.4 Correlations between cognitive emotion regulation strategies and emotional manipulation

**Table 2** shows the Pearson correlation coefficients for the relationship between the mean overall score of emotional manipulation and the CER strategies. The emotional manipulation score had a small but statistically significant negative correlation with two adaptive emotional regulation strategies: positive refocusing, r = -0.146, p = 0.005 and focus on planning, r = -0.144, p = 0.006.

## 4 Discussion

This study examined the relationship between CER strategies and emotional manipulation among undergraduate students at health colleges of a public university in Riyadh, Saudi Arabia, with a total sample size of 362 students. Data were collected using two standardized reliable questionnaires, the emotional manipulation scale and the CER questionnaire. The internal reliability of the CER questionnaire and the emotional manipulation scale was 0.75 and 0.767, respectively, supporting its internal consistency among the study sample. The age range of the participants was 18 – 22 years. Most of them had a very good academic GPA of ≥3.75 out of 5 and no mental health illness.

### 4.1 Emotional manipulation

There was a moderate level of emotional manipulation among the respondents. A previous meta-analysis showed that sex had a moderating effect on the relationship between self-reported ability-based emotional intelligence and non-prosocial emotional manipulation, where this relationship was stronger among males than among females (13). Our study has a homogeneous sample of undergraduate female students that increases its value by controlling the effect of sex variations on the sample.

Emotional manipulation had a strong positive correlation with personality traits such as narcissism, Machiavellianism, and psychopathy, especially among men compared to women (26). Grandiose and vulnerable narcissists are prone to emotionally manipulate others to achieve their intended goals (27). A previous study showed that emotional manipulation had a positive association with moral disengagement (28), which proved the argument that emotional manipulators have no morals (29). Therefore, people who engage in emotional manipulation are more likely to use moral disengagement to justify their unethical or illegal behavior (28). In addition, moral disengagement mediated the relationship between emotional manipulation and psychological well-being (28). Therefore, emotional manipulation could potentially put students of health colleges at risk of compromised well-being and unethical behavior. Training students to be emotionally intelligent without manipulating others is critical not only for their safety but also for their psychological health.

Emotional manipulation has been considered a harmful trait in people with high emotional intelligence (27, 30). Following the theory of mind, it has been claimed that emotional manipulation will not occur without understating the emotions of others to easily influence their reactions (13). Consequently, these people who manipulate others might use specific cognitive emotional strategies to achieve their effects. However, the strategies that students use to manipulate others have not been fully examined, which drives the current study to fill this literature gap.

### 4.2 Cognitive emotional regulation strategies

The study revealed the students’ preferred strategies for managing their emotions. The most frequently used adaptive cognitive strategy was positive reappraisal, which means that students give a positive meaning to stressful events in terms of personal growth (23). While the least used adaptive strategy was positive refocusing, which means that the students are not able to shift their thinking from the actual event to think about enjoyable things (23), which may have a negative impact on their mental health. On the other hand, the most frequently used maladaptive strategy was rumination (thinking about feelings and thoughts related to negative events), while blaming others (thinking about blaming others for their own problems) was the least used strategy. These results suggest that the students were aware of the regulation of their emotions through a healthy coping mechanism, such as positive reappraisal. However, they still need training on how to use other cognitive strategies, such as positive refocusing, as well as how to reduce the rumination strategy.

The positive relationship between cognitive emotion regulation and mental health status in terms of anxiety and depression has been studied in adolescents (31, 32). Increasing self-blame, catastrophizing, and rumination, as well as reducing positive refocusing and acceptance, were associated with higher symptoms of anxiety or depression (33). The ability of nurses to control their emotions and work performance has been found to be related to their adopted CER strategies (34, 35). Therefore, it is necessary to equip health colleges students to manage their emotional challenges effectively through a potential training area such as shifting negative thoughts to more positive thoughts. By understanding how the students regulate their emotions and which strategies they use and do not use, the educators can design a tailored intervention to promote their use of adaptive strategies that can enable them not only to manage their emotions more effectively, but also to improve their overall psychological well-being. It is important to promote healthy emotional regulation strategies by designing interventional workshops that equip students with a variety of adaptive strategies, such as positive refocusing techniques. Cognitive behavioral therapy and mindfulness interventions can be particularly helpful in enhancing self-awareness and emotional regulation skills, reducing dependency on maladaptive strategies, and fostering more effective coping mechanisms.

### 4.3 Correlations between cognitive emotion regulation strategies and emotional manipulation

The total emotional manipulation score had a small but statistically significant negative correlation with two adaptive emotional regulation strategies: positive refocusing and focus on planning. These correlations suggest that decreased positive refocusing and focus on planning may increase participants’ tendency to emotionally manipulate others.

To our knowledge, this study is the first to investigate the correlation between CER strategies and emotional manipulation. The overall emotional manipulation score had a statistically significant negative correlation with two adaptive emotional regulation strategies (positive refocusing and focus on planning). These correlations suggest that decreased positive refocusing and focus on planning may increase participants’ tendency to emotionally manipulate others. Research has shown the effectiveness of CER in modifying emotional responses, especially in stressful situations (36). Interestingly, Raio and colleagues found that stress could significantly hinder the effectiveness of emotional regulation techniques (36). The study participants are students at health colleges, which means they might be exposed to a high level of stressful situations. Therefore, stress might be a mediator between CER strategies and emotional manipulation. Other studies should examine this relationship and the mediating effect of stress, as it is not yet proven. It is worth noting that 17.65% of the participants in our study reported having a history of mental health conditions. This suggests that university students are in need of continuous psychological support. A future study is required to explore the relationship between the mental health status of students and the CER strategies used.

### 4.4 Implications of the study

The study has many implications for future research. For instance, the study added to the literature by shading the light for the first time, to our knowledge, on the relationship between CER strategies and emotional manipulation, with recommendations to explore other factors that could cause healthcare workers to manipulate others. Two cognitive coping strategies have shown a correlation with emotional manipulation should be considered by the educators to provide academic support to students, especially in improving their skills in positive refocusing and focus on planning.

This study emphasises the significance of developing educational programs that intervene to assist students at health colleges in effectively managing their cognitive emotional coping strategies. Thus, equip students with the skills to effectively navigate their lives and prevent emotional manipulation, both for their own well-being and the well-being of others. Furthermore, the results of this study can assist future researchers formulate hypothesis and conduct further investigations in this topic.

### 4.5 Limitations and recommendations

Although our study contributes to the existing literature on CER and emotional manipulation among university students, it is important to acknowledge its limitations to be considered in future studies. First, this study adopted the cross-sectional design that examined the correlation using the univariate analysis method, which might limit the development of a causal relationship between the studied variables. Therefore, a longitudinal study is necessary. A future longitudinal study is needed to see whether work experience, place of work, managerial support, and patients’ attitudes can change students’ behavior and might lead to changes in CER and emotional manipulation attitudes. Second, the study sample was recruited from a single university, therefore a multi-settings study is required in future studies. Although a large number of undergraduate students from various health colleges were invited to participate, the findings may not apply to students in other colleges with different contexts. Third, another limitation that should be considered in future studies is that our study includes only females, which makes it impossible to generalize to males. However, this study serves as a foundation for future research to assist in formulating inferences and training programs to improve CER among students. Fourth, the self-reporting questionnaire may cause response bias, although we used standardized and validated questionnaires. It is essential to clarify that the questionnaire was used in English among our sample, since English proficiency is a mandatory admission requirement for health colleges in Saudi Arabia. However, a future psychometric study among Arabic speaking is required. Fifth, additional limitation is that 17.6% of the study participants reported having mental health illness. This should be considered when interpreting the findings, as mental health conditions may influence emotional manipulation and CER. Future studies could explore the specific relationship between mental health issues and CER and emotional manipulation.

## Data Availability

The raw data supporting the conclusions of this article will be made available by the authors, without undue reservation.
